# Experimental Study on Magnetic Coupling Piezoelectric–Electromagnetic Composite Galloping Energy Harvester

**DOI:** 10.3390/s22218241

**Published:** 2022-10-27

**Authors:** Xia Li, Tongtong Ma, Benxue Liu, Chengming Wang, Yufeng Su

**Affiliations:** School of Mechanical and Power Engineering, Zhengzhou University, Zhengzhou 450001, China

**Keywords:** piezoelectric energy harvester, composite energy harvester, wind-induced vibration, magnetic coupling, galloping energy harvester

## Abstract

In order to solve the demand for low-power microcomputers and micro-electro-mechanical system components for continuous energy supply, a magnetic coupling piezoelectric–electromagnetic composite galloping energy harvester (MPEGEH) is proposed. It is composed of a piezoelectric energy harvester (PEH) and an electromagnetic energy harvester (EEH) coupled by magnetic force. The bistable nonlinear magnetic coupling structure improves the output power of the MPEGEH. The advantages and output performance of the MPEGEH are analyzed. The prototype of the energy harvester is made, and the nonlinear output characteristics under different load resistances are analyzed. Through the experiment on the key parameters of the composite energy harvester, it is found that the higher the coupling degree of the two parts of the MPEGEH, the stronger the nonlinear characteristics and the better the output characteristics. The results show that the onset wind velocity and output power of the MPEGEH are better than the classic galloping piezoelectric energy harvester (CGPEH). At the same wind speed, with the increase in the distance d_0_ between magnets A and B, the output power of both the PEH and the EEH decreases. When d_0_ is 37 mm, the output power of the EEH is the largest. The distance s_0_ between magnets B and C has little influence on the output power of the PEH but has a great influence on the EEH. When s_0_ is 23 mm, the EEH has the best output characteristics. Compared with the CGPEH, the onset wind velocity is reduced by 28%, and the output power is increased by 136% when the wind speed is 11 m/s.

## 1. Introduction

With the rapid development of the Internet of Things industry, there is more and more demand for products such as wireless sensors and microelectronics systems, and their requirements are getting higher and higher. Most of these devices work in a poor environment and cannot have their batteries replaced frequently. The wired power supply also greatly limits their portability and freedom of application. Therefore, the continuous supply of low-power equipment has become a hot issue for researchers at home and abroad. Vibration energy collection technology can effectively capture energy from the working environment of equipment and has developed rapidly in the past 20 years. At present, the common modes of vibration energy capture are piezoelectric [1,2], electromagnetic [3,4], triboelectric [5], and thermoelectric [6]. Among them, the piezoelectric energy harvesting device has the advantages of simple structure, high output voltage, wide working frequency range, and high energy density [7,8,9], Additionally, the electromagnetic energy harvesting device has the advantages of simple structure, large output current, and high energy collector efficiency [10]. Both are suitable for power supply to wireless sensors and micro-electrical systems [11,12,13,14], which have received widespread attention from domestic and foreign researchers.

The wind-induced vibration is very common, collecting energy from the vibration of wind is one of the key directions of the current research. The devices that collect energy from the vibration of the wind are mainly based on vortex-induced [15,16,17], galloping-induced [18,19,20], flutter-induced [21,22], and wake-induced [23,24]. Gallop is a kind of self-excited divergent vibration that arises from the influence of the non-streamlined cross-sectional structure with prisms. It has the characteristics of large amplitude and large deformation, the operating frequency is wider, and the energy capture effect is more obvious. Thus, miniature wind energy harvester-based gallops have received extensive attention from researchers. Many researchers’ research directions are concentrated on the impact of changes in the main structure of the captive energy device on their output characteristics and the modeling analysis of the modeling of relaxation capture energy devices. Soltanrezaee et al. [25] studied the effects of the isosceles triangle tip body with different apex angles on the energy trapping effect of the energy harvester. Additionally, the effects of piezoelectric ceramic number, size, and position on the energy harvester’s output characteristics were experimentally verified. It improved its output power but ignored the power density of the captive energy device. Zhao et al. [26] used single-freedom models, single-mode and multi-mode Eura-Bernubari distributed parameter models, and other modeling methods to study the effects of load resistance, bluff body volume and mass, and piezoelectric sheet length on the output power of the energy harvester. The onset wind speed and aeroelastic behavior of the energy harvester structure are well predicted. Zdenek et al. [27] use a single degree-of-freedom (DOF) model to compare the energy in the energy collection operations (PZT-5A bimorph, PZZN-PLZT bimorph, and PVDF unimorph). At the same time, the influence of the modal shape function on the simulation results is analyzed. Through comparison, it is found that PZT-5A and PZZN-PLZT are excellent choices for energy collection due to their high-power output. Dunnmon et al. [28] used the nonlinear limit cycle oscillation of an aeroelastic energy collector whose cantilever is connected to the trailing edge of a fixed-wing to conduct a numerical study on the oscillation characteristics and collection performance of the limit cycle oscillation, and the output effect obtained is consistent with the experimental results. Javed et al. [29] studied the influence of different air loads on the gallop and proposed different expressions of relaxation vibration. The influence of electric load resistance on the performance of the harvester under different aerodynamic forces is found through parameter research.

In order to improve the output characteristics of the energy harvester, some researchers have innovated the structure of the bluff body. Alhadidi et al. [30] added Y-shaped fins on the back surface of the bluff body of the galloping energy harvester to improve the sensitivity of the energy harvester and reduce the response time. Compared with the fin-free structure, the response time is shortened by 75%, and the stability of the galloping energy harvester is not affected. Abdelkefi et al. [31] established a nonlinear parametric model to discuss the influence of different cross-sectional geometries such as D-shape, square, and isosceles triangle on the power of the energy harvester under different loads and wind speeds. The results show that the cross-sectional geometry of the bluff body has a significant effect on the initial velocity and the maximum power at different wind speeds. The energy harvester with triangular and square bluff body sections has the maximum output power at low wind speeds. When the wind speed is high, the output power of the D-shaped blunt body energy harvester is the best.

The optimization of the structure of the energy harvester device has improved its energy harvester efficiency and broadened the working frequency band to a certain extent. However, in order to meet the increasing energy demand and more complex working conditions of the microelectronic system, some scholars have carried out relevant research on the composite energy harvester device [32]. Challa et al. [33] added an electromagnetic component on the bluff body side of a piezoelectric energy collection device to better match the total damping and mechanical damping in the system, so that the peak output power of the piezoelectric part can be increased by 30%. The total power output is increased by 65.5% compared with the corresponding independent single-collection mode device. Su et al. [34] introduced magnetic force into the design of a bi-directional U-shaped piezoelectric energy harvester for vortex-induced vibrations. The theoretical model of the beam structure is derived based on the Euler–Bernoulli beam theory. After adding magnets to the system, significant improvement of the lock-in region and the peak voltage are noticed in the horizontal mode under both up and down sweeps. Toyabur, salauddin et al. [35] verified experimentally that a hybrid piezoelectric electromagnetic energy harvester with multiple mechanical degrees of freedom based on multi-mode can work in four resonance modes in the range of 12–22 Hz. Under 0.4 g acceleration and 90 KΩ load, the maximum power is 250.23 µW in the third resonance mode. The output power is increased, and the working frequency band of the energy trap is greatly widened. Ferrari et al. [36] fixed a permanent magnet on the cantilever beam and fixed an external permanent magnet with opposite polarity on the micrometer table. Designed a nonlinear dual-stable captive energy structure. Establish a Duffing equation for dual-stabilized cantilever beams and study the impact of nonlinear items on the dual stability phenomenon. Analyses and simulations show that a nonlinear bistable converter gives better performances under wideband excitation with respect to a linear system. Stanton et al. [37] further studied the bistable piezoelectric power generation structure and obtained the coexistence of multiple attractors, chaos, large amplitude oscillation, and other phenomena.

Most of the research on the composite energy harvester focuses on the excitation mechanism, and the research on the coupling of the galloping piezoelectric energy harvester and the electromagnetic energy harvester is less. This article proposes a magnetic coupling composite energy harvester The bistable nonlinear magnetic coupling can improve the energy harvester characteristics of the relaxation piezoelectric energy harvester device and further improve its output performance. At the same time, the superposition of an electromagnetic energy harvester can not only greatly improve the output power but also broaden the working frequency band of the composite energy harvester. The magnetic coupling piezoelectric–electromagnetic composite galloping energy harvester (MPEGEH) can continuously provide energy for sensors and electronic equipment with more complex working environments. In this paper, the coupling characteristics of the MPEGEH are analyzed, the structure of the electromagnetic energy harvester (EEH) is designed, its working principle is analyzed, and the experimental study on the output characteristics of the composite energy harvester is completed.

## 2. Structure Design of the MPEGEH

### 2.1. Modeling and Manufacturing

**Figure 1:**
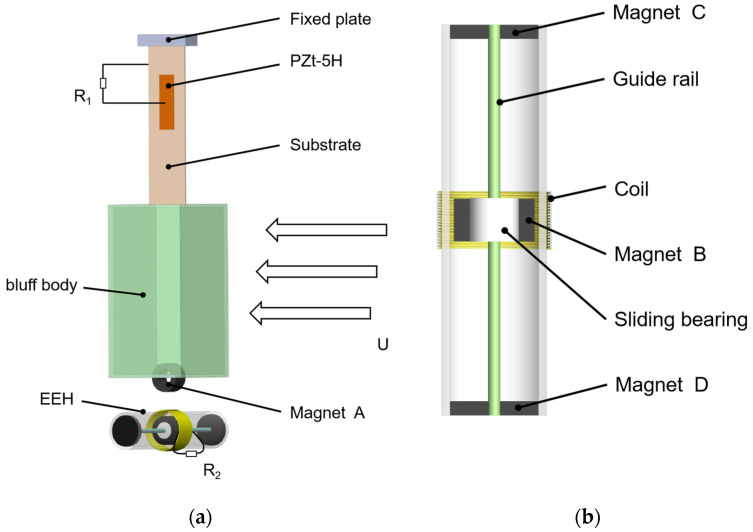
Structural diagram of the MPEGEH: (**a**) The MPEGEH general structure schematic diagram; (**b**) the EEH structure schematic diagram.

The MPEGEH is composed of the PEH and the EEH coupled by magnetic force. As shown in Figure 1a.

The PEH consists of a copper cantilever beam, a PZT-5H piezoelectric sheet [38], and a rectangular blunt body. The piezoelectric sheet is pasted on the fixed end of the cantilever beam through conductive silica gel, and the blunt body is installed on the free end of the cantilever beam. When the wind speed exceeds the starting wind speed of the MPEGEH, the energy absorbed by the energy harvester from the environment is greater than the energy consumed by the vibration, and the blunt body generates a lateral gallop [39]. The piezoelectric sheet is connected to the load resistance R_1_ to convert vibration energy into electric energy.

The EEH consists of a sleeve, the magnet B, a coil, a guide rail, magnets C and D, etc. As shown in Figure 1b. The sleeve is fixed on the clamp, and magnets C and D are fixed at the ends of the sleeve. The magnet B is connected to the guide rail by a sliding bearing. The coil and the sleeve are fixed on the central surface of the sleeve on the same axis. When the blunt body vibrates, magnet A drives magnet B to reciprocate in the sleeve to cut the magnetic sensing line. The coil is connected to load resistance R_2_ to convert kinetic energy into electric energy. The direction of magnets C and D magnetic poles is the opposite of magnet B. The magnetic B nonlinear recovery power is given through the Repulsive force of each other.

### 2.2. Working Principle of the MPEGEH

The airflow contacts the bluff body along the positive direction of the *Z*-axis, and the negative aerodynamic damping caused by the flow separation and vortex shedding causes the wind-induced galloping of the energy harvester. This causes the cantilever beam to oscillate periodically and drives PZT-5H to reciprocate [40,41]. Based on the positive piezoelectric effect, the potential difference between the upper and lower surfaces of PZT-5H forms a complete current loop with external wires and resistors [42]. Magnet A swings together with the blunt body and drives magnet B in the sleeve to reciprocate along the guide rail direction under the restoring force provided by the magnetic spring. Magnet B repeatedly cuts the magnetic induction line of the coil [43]. According to the law of electromagnetic induction, alternating current will be generated in the coil to power the load resistance R_2_.

## 3. Experimental Device

**Figure 2:**
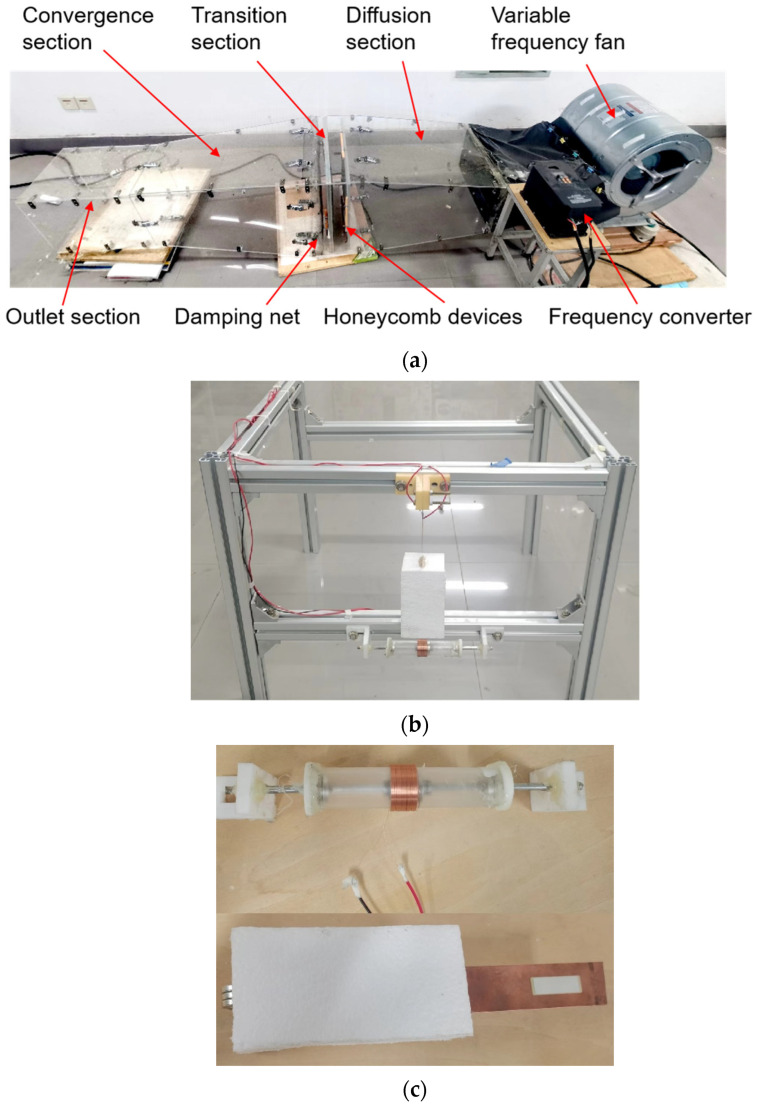

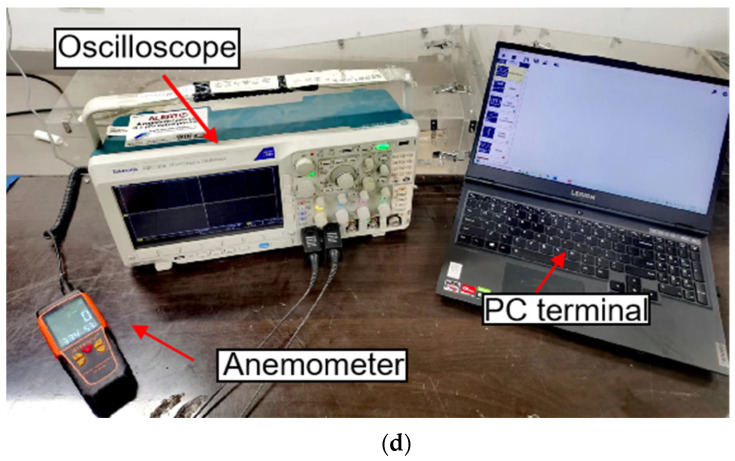
Experimental equipment: (**a**) wind tunnel device; (**b**) energy collection system; (**c**) experimental models of the EEH and the PEH; (**d**) data acquisition and processing system.

The experimental device is composed of the wind tunnel experimental system, the energy collection system, the data collection, and the processing system, as shown in Figure 2.

Figure 2a shows the simple wind tunnel used in the experiment. Its sectional dimension is 300 mm × 300 mm and is mainly composed of a diffusion section, transition section, contraction section, and outlet section. Additionally, a damping net and two honeycomb devices installed in the transition section ensure uniform and stable air flow in the outlet section. During the experiment, the variable frequency fan (DWF 3.15L, Shandong kepuda fan Co., Ltd., Dezhou, China) was adjusted by the frequency converter (V8 4T 4R0GB, Shenzhen Weike Technology Electronics Co., Ltd., Shenzhen, China) to control the wind speed in the wind tunnel.

The energy collection system is composed of the MPEGEH and a base clamp, as shown in Figure 2b. Figure 2c shows the experimental models of the EEH and the PEH. As shown in Figure 2d, the data acquisition and processing system is composed of an oscilloscope (MDO 3014, Tektronix Inc., Beaverton, OR, USA), an anemometer (AS-H3, Wuhan Zhongce bentu Measuring Instrument Co., Ltd., Wuhan, China), and a PC terminal. It can measure, display, and record the wind speed and output voltage of the energy harvester in real-time. The elongation of the copper substrate material used in the experiment is 30%, the tensile strength is 315 MPa, and the shield material is 25 K high-density foam. With reference to the theoretical model of the relaxation energy trap built by Li et al. [42], the size of the actual experimental platform and the power of the inverter fan, the structural parameters of the MPEGEH are tentatively determined as shown in Table 1.

**Figure 3:**
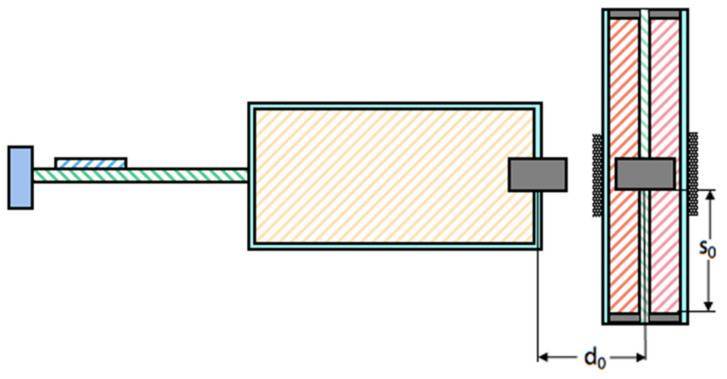
The MPEGEH cross section.

During the experiment, the initial distance d_0_ between magnets A and B is set to 40 mm, the static distance s_0_ between magnets B and C is set to 25 mm, and the same is true between magnets B and D, as shown in Figure 3. The load resistance R_1_ is tentatively set to 5 × 10^5^ Ω, and the load resistance R_2_ is tentatively set to 35 Ω.

## 4. Experiment and Discussion

In order to test the energy trapping effect of the MPEGEH and study its output characteristics, this chapter conducts experiments by matching different load resistances and adjusting the key parameters d_0_ and s_0_ of the energy harvester. The influence of load resistance and key parameters of MCPEE on its output characteristics is analyzed, and the reasonable distance between magnets and the matching resistance is determined.

### 4.1. Experimental Study on Output Performance of the MPEGEH

When the wind flows through the energy harvester, under the action of the edges of the rectangular bluff body, the bluff body appears regularly unstable. When the wind speed exceeds the bluff body, it will generate a vortex and fall off, forming an aerodynamic negative damping component, which reduces the critical speed of the energy harvester and remains stable, and the motion form of the MPEGEH changes to galloping with constant amplitude. At this time, the voltage generated by PZT-5H is proportional to the strain and stress of the substrate. Therefore, the aeroelastic response of the energy harvester can be quantitatively analyzed. In this paper, the output voltage and output current are used to evaluate the vibration response characteristics.

**Figure 4:**
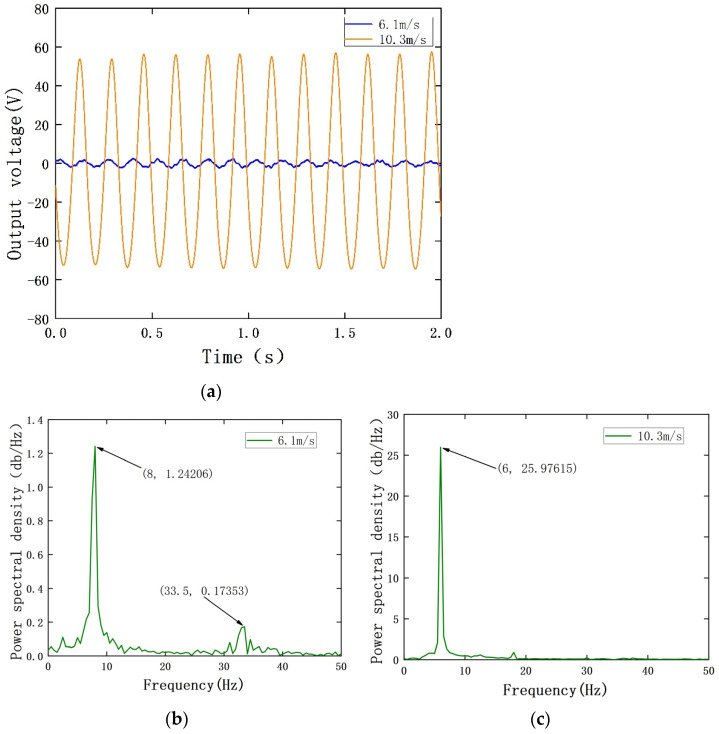
The output performance of the PEH under different wind speeds: (**a**) the PEH output power at different wind speeds; (**b**) output power spectrum density of the PEH at 6.1 m/s; (**c**) output power spectrum density of the PEH at 10.3 m/s.

**Figure 5:**
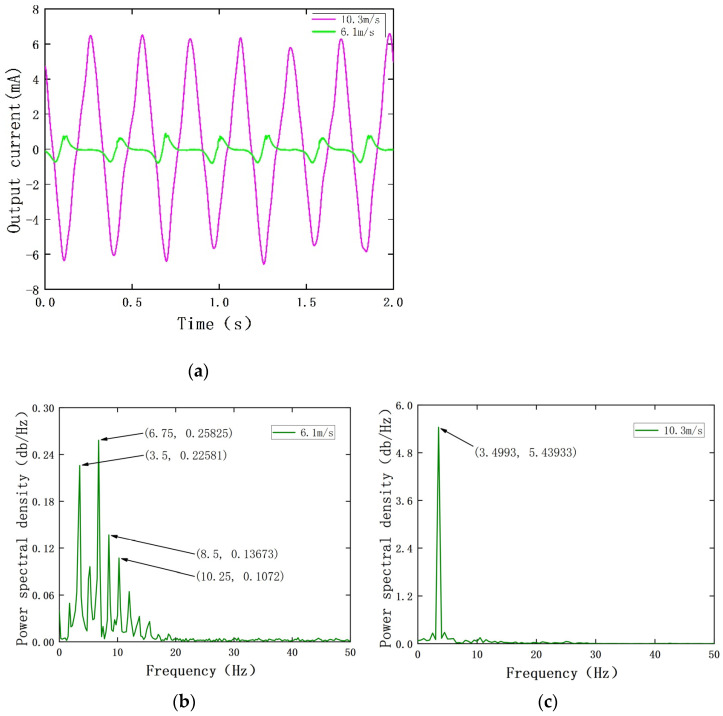
The output performance of the EEH at different wind speeds: (**a**) output voltage of the EEH; (**b**) output power spectrum density of the EEH at 6.1 m/s; (**c**) output power spectrum density of the EEH at 10.3 m/s.

When the wind speed is between 6.1 m/s and 10.3 m/s, the measured output voltage and power spectral density of the PEH are shown in Figure 4. Additionally, the measured output current and power spectral density of the EEH are shown in Figure 5. During the experiment, in order to avoid plastic deformation of the substrate or the excessive amount of PZT-5H deformation, the MPEGEH caused permanent damage; the maximum wind speed used was 11 m/s.

It can be seen from Figure 5a that when the wind speed is 6.1 m/s, the oscillation frequency and amplitude of the magnet driven by the blunt body of the composite energy harvester are too small. This causes the movement of magnet B to be intermittent, and the movement distance is extremely short. As shown in Figure 5b, the vibration response frequency is repeated between 0 and 16.5 Hz, and the current generated by the EEH cannot be utilized. When the wind speed is 10.3 m/s, magnet B moves periodically under the influence of magnet A to cut the magnetic induction line. The current in the circuit is 6.5 mA, and the power spectral density is shown in Figure 5c.

**Figure 6:**
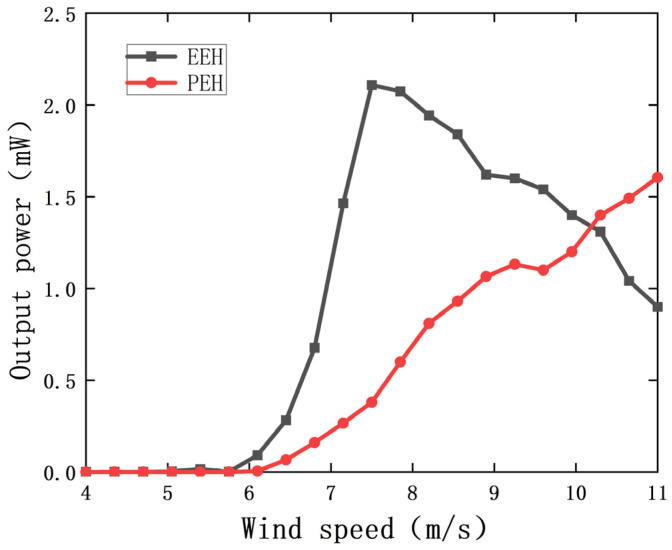
Output power curve of the PEH and the EEH with wind speed.

Figure 6 shows the output power curves of the PEH and the EEH under different wind speeds. It can be seen from the figure that the starting wind speed of the PEH is 6.75 m/s. After reaching the turning wind speed, the output power of the PEH increases with the increase in wind speed. After the PEH is vibrated, the EEH is quickly vibrated and reached the maximum power value, and the output power will gradually decrease with the increase in wind speed. When the wind speed is 11 m/s, the total output power of the MPEGEH is 2.5 mW, of which the PEH and the EEH output power is 1.6 mW and 0.9 mW, respectively.

### 4.2. The Effect of Load Resistance on the Output Performance

**Figure 7:**
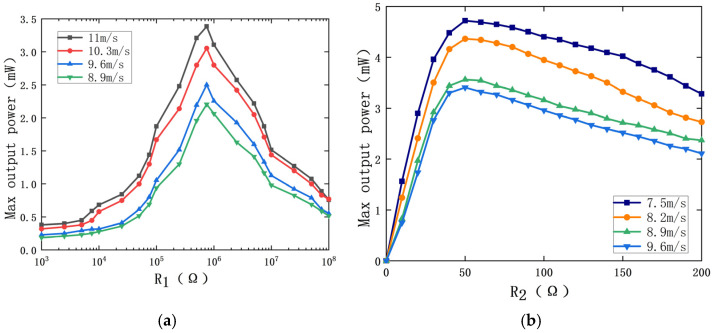
The effect of load resistance on the MPEGEH output power at different wind speeds: (**a**) effect of R_1_ on the PEH output power; (**b**) effect of R_2_ on the EEH output power.

In order to study the maximum output power of the MPEGEH, it is necessary to analyze its optimal matching load. In order to specifically determine the value of the best load resistance of the PEH, the load resistance R_2_ is tentatively set to 35 Ω, and the variation of the output power of the PEH with load resistance R_1_ at different wind speeds (11 m/s, 10.3 m/s, 9.6 m/s, and 8.9 m/s) is studied, as shown in Figure 7a. It can be seen from the figure that under the same wind speed, the maximum output power of the PEH increases with the increase in the load resistance R_1_ at first, and decreases with the increase in the resistance after reaching the maximum value, showing a normal distribution on the whole. The wind speed is 11 m/s and the load resistance R_1_ is 7.5 × 10^5^ Ω, the maximum output power of the PEH is 3.5 mW. When the wind speed is 10.3 m/s, 9.6 m/s, and 8.9 m/s, it also reaches its peak at 7.5 × 10^5^ Ω. Therefore, 7.5 × 10^5^ Ω is the optimal value of the load resistance of the PEH.

Keep the load resistance R_1_ at 7.5 × 10^5^ Ω and change the size of load resistance R_2_ to study the effect of load resistance R_2_ on the output power of the EEH. When the wind speed is 9.6 m/s, 8.9 m/s, 8.2 m/s, and 7.5 m/s, the change of the EEH maximum output power with load resistance R_2_ is shown in Figure 7b. From Figure 7b, it can be seen that the output power of the EEH is generally nonlinear with the change of R_2_. When the load resistance R_2_ is constant, the maximum output power increases with the increase in wind speed. When the wind speed is certain, the maximum output power of the EEH first increases with the increase in the load resistance, and the increase is faster. When R_2_ is 50 Ω, the output power of the EEH reaches its peak and then gradually decreases, but the reduction trend is slow. Therefore, 50 Ω is the optimal value of the EEH load resistance.

### 4.3. Influence of Key Parameters on Output Characteristics of the MPEGEH

#### 4.3.1. The Distance d_0_ between Magnets A and B

**Figure 8:**
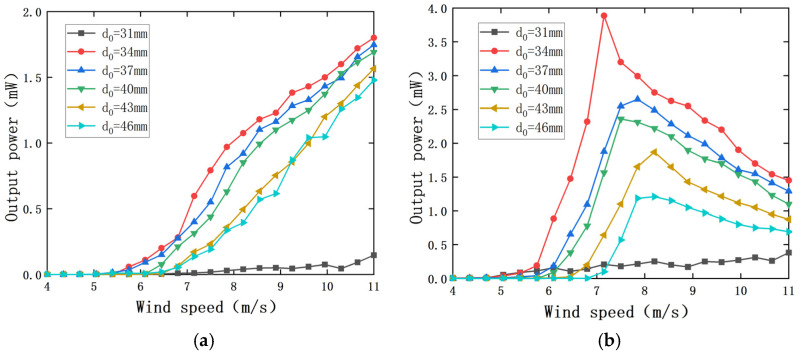
Change curve of output power with wind speed under different d_0_: (**a**) output power of the PEH; (**b**) output power of the EEH.

The distance d_0_ between magnet A and magnet B is an important factor affecting the coupling effect of the PEH and the EEH, which will greatly affect the output characteristics of the two parts. The diameter of magnet A is 10 mm, and the outer diameter of the coil is 29 mm, so the d_0_ should not be less than 19.5 mm. The load resistance R_1_ of the PEH is 7.5 × 10^5^ Ω, and the load resistance R_2_ of the EEH is 50 Ω. The impact of d_0_ on the output characteristics of the captive energy harvester is shown in Figure 8.

It can be seen from Figure 8 that there is a reasonable size range for the magnet spacing d_0_. When d_0_ is less than 34 cm, the change in wind speed has little impact on the output power of the PEH and the EEH. When d_0_ is greater than or equal to 34 cm, the influence of wind speed on the output power of the PEH and the EEH is nonlinear. In the range of experimental wind speed, with the increase in magnet spacing d_0_, the maximum output power of the PEH and the EEH decreases, and the starting wind speed increases.

When d_0_ is 31 mm, the output power of the PEH and the EEH does not change significantly with the increase in wind speed. This is because when the distance between the magnet and magnet B is too close, a strong interaction force will be generated between the two magnets, and the PEH and the EEH are in a very strong nonlinear coupling state. The aerodynamic force generated by the wind speed used in the experiment cannot drive the bluff body to have a regular instability phenomenon and cannot achieve effective energy capture.

When d_0_ is greater than or equal to 34 cm, the experimental wind speed range is 34 mm~46 mm. With the increase in d_0_, the wind speed of the composite energy harvester increases. When the wind speed is greater than the starting wind speed, the output power of the PEH increases nonlinearly with the increase in wind speed and decreases with the increase in magnet spacing d_0_ at the same wind speed. The output power of the EEH changes in a normal distribution with the wind, and the output power decreases with the increase in the magnet spacing d_0_ at the same wind speed. The starting vibration wind speed (7.2 m/s) when d_0_ is 46 mm is 1.26 times the starting vibration wind speed (5.7 m/s) when d_0_ = 34 mm. When the wind speed is 11 m/s, the output power of the PEH is 1.8 mW when the magnet spacing d_0_ is 34 mm, which is 1.29 times that when d_0_ is 46 mm (1.4 mW), and the output power of the EEH is 1.45 mW when the magnet spacing d_0_ is 34 mm, which is 2.1 times that when d_0_ is 46 mm (0.69 mW). It can be seen that the magnitude of d_0_ has a significant effect on the output power of both the PEH and the EEH. The magnet spacing d_0_ has a great influence on the output power of the EEH because the magnetic force between magnet A and magnet B is the only driving force of the EEH, and its output characteristics are greatly affected by d_0_. According to the above analysis, it is determined that the distance d_0_ between magnets A and B in this experiment is 34 mm.

It can be seen from Figure 8 that when d_0_ is set to 37 mm, 40 mm, 43 mm, and 46 mm, respectively, the output power of the PEH and the EEH changes relatively smoothly with the distance d_0_ between magnets A and B. However, when d_0_ is 34 mm, the power output curve increases by leaps and bounds at 5.75 m/s. At 7.15 m/s, the output power of the EEH reached 3.9 mW and then slowly decreased as the wind speed increased. In order to analyze the cause of the jump in output power, the wind speed before and after the jump in the PEH and the EEH is analyzed when d_0_ is 34 mm. The wind speed before the jump is 6.15 m/s and 6.65 m/s, and the wind speed after the jump is 7.65 m/s and 8.15 m/s.

**Figure 9:**
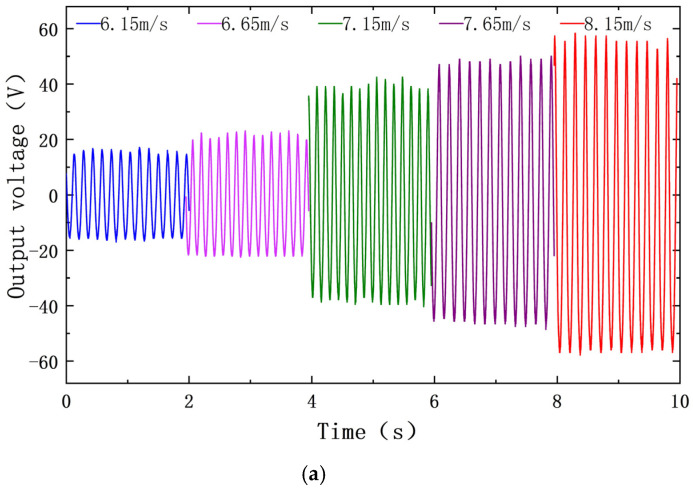

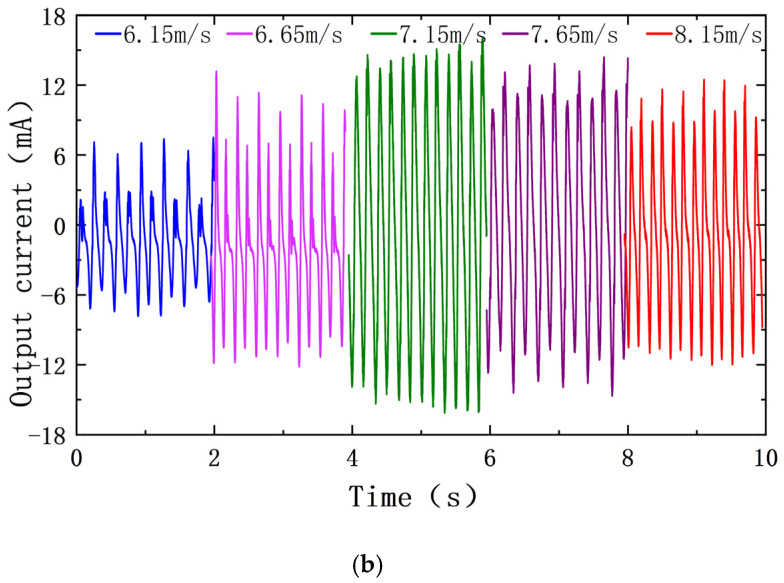
Output time-domain curve under different wind speeds before and after the jump at d_0_ is 34 mm: (**a**) the PEH output voltage; (**b**) the EEH output current.

Figure 9a,b show time-domain curves of output voltage and current of the PEH and the EEH under the above five wind speeds, respectively. It can be seen from Figure 9a that the output voltage of the PEH is relatively stable before and after the jump, which is similar to the sine function graph. Figure 9b shows that the output current of the EEH is chaotic before the jump, and the waveform tends to the sinusoidal function graph after the current jump. This shows that the vibration form of the EEH changes obviously before and after the jump.

**Figure 10:**
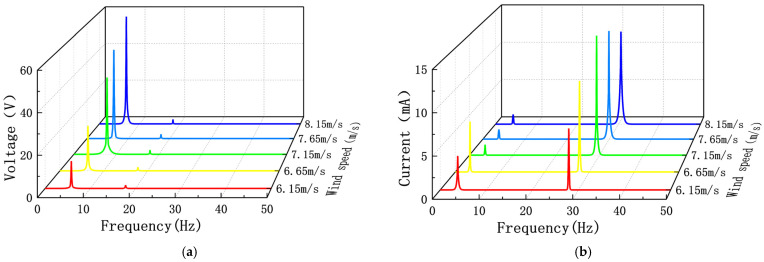
Fourier transform curve under different wind speeds before and after the jump at d_0_ is 34 mm (**a**) the PEH (**b**) the EEH.

Figure 10 shows the Fourier change curve of the output voltage and output current of the PEH and the EEH when d_0_ is 34 mm. From Figure 10, it can be observed that the PEH has no significant changes except for changes in the voltage of the amplitude-frequency curve before and after the jump change. The vibration is a single-frequency vibration corresponding to the first main frequency. The amplitude-frequency curve of the EEH before the jump is a multifrequency form that appears at the same time at the same time of the first and second main frequencies. Near the jump point, the first main frequency range value is reduced. This phenomenon often occurs in strongly nonlinear systems. The vibrator of the EEH is affected by the nonlinear magnetic force of two magnetic springs and a moving permanent magnet. The magnitude of d_0_ affects the magnitude of the nonlinear force of the permanent magnet on the vibrator. The decrease in d_0_ causes the vibrator to exhibit stronger nonlinear characteristics. In the nonlinear system, the nonlinear natural frequency is related to the vibration amplitude. With the increase in the vibration amplitude, the natural frequency also increases, and the amplitude jump occurs at the frequency corresponding to the maximum displacement response.

#### 4.3.2. The Influence of the Distance s_0_ between Magnets B and C

**Figure 11:**
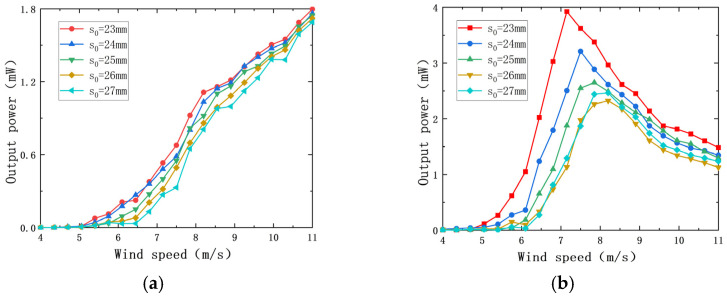
The variation of the output power with wind speed at different s_0_: (**a**) the PEH output power; (**b**) the EEH output power.

Magnet C and magnet D act as magnetic springs to provide the only restoring force for the movement of magnet B. The distance s_0_ between the magnets B and C directly affects the output characteristics of the EEH. The curve of the EEH and the PEH output power varies with wind speed at different sizes s_0_ is shown in Figure 11. During the experiment, d_0_ was 34 mm. From Figure 11a, we can observe that the change of s_0_ only has a little effect on the output power of the PEH. When the wind speed is 11 m/s and s_0_ is 23 mm, the output power of the PEH is 1.79 mW, which is 5.9% higher than the output power (1.69 mW) when s_0_ is 27 mm at the same wind speed. For the EEH, it can be seen from Figure 10b that when s_0_ is 23 mm, there is an obvious jump in the curve of output power with time relative to the four groups where s_0_ is 24, 25, 26, and 27 mm. When the wind speed is 7.15 m/s, the output power reaches 3.92 mW.

**Figure 12:**
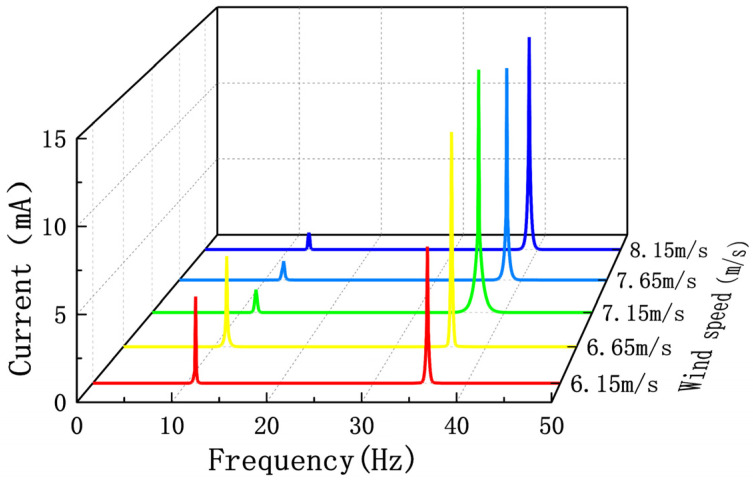
Fourier transform curves of the EEH output current at different wind speeds before and after the jump at s_0_ is 23 mm.

When s_0_ is 23 mm, wind speeds of 7.0 m/s and 6.5 m/s were selected before the EEH jump, and wind speeds of 8.0 m/s and 8.5 m/s were selected after the EEH jump to analyze the jump phenomenon. Figure 12 is the FFT curve of its output current. From Figure 12, it can be seen that when the wind speed is before the jump, the frequency of the output current is mainly 12 Hz and 31 Hz. After the jump, the frequency of the output current is mainly single frequency. This shows that smaller s_0_ will make the vibration and output of the EEH have strong nonlinearity. At the same time, it also makes the output power of the EEH have a high output power within a specific wind speed range.

## 5. Comparison with Classical Galloping Energy Harvester

**Figure 13:**
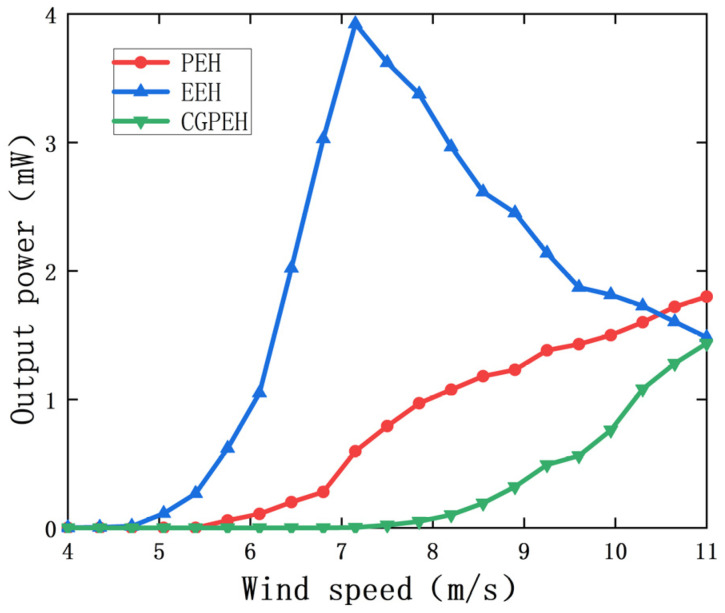
Output power change curve of the PEH, the EEH, and the CGPEH with wind speeds.

In order to verify the output performance of the MPEGEH, a classic galloping piezoelectric energy harvester (CGPEH) with the same structural parameters as the MPEGEH was fabricated and compared. Figure 13 shows the output power curves of the PEH, the EEH, and CGPEH. It can be seen from Figure 13 that the starting wind speed of the PEH is 5.4 m/s, which is 28% lower than that of CGPEH (7.5 m/s). When the wind speed is 11 m/s, the output power of the PEH and the EEH is 1.79 mW and 1.48 mW, respectively, and the total output power of the MPEGEH is 3.27 mW, which is 229% higher than that of the CGPEH (1.43 mW). Therefore, in the MPEGEH, the dynamic nonlinear magnetic coupling between the PEH and the EEH can effectively reduce the starting wind speed of the energy harvesting device and significantly improve the output power of the energy harvesting device. This proves the superiority of the MPEGEH performance.

## 6. Conclusions

In order to improve the output characteristics of the energy harvester and improve the output power of the energy harvester, so that it can continue to provide energy for microelectronic components in a more complex working environment. This article proposes a magnetic coupling conformal piezoelectric–electromagnetic composite energy harvester based on wind-induced galloping. The geometric model is established, relevant experiments are completed, and the coupling of output performance between the PEH and the EEH is analyzed. The influence of key parameters on its output performance is analyzed and compared with the output performance of the classical galloping energy harvester. The specific research results are as follows:

(1) A piezoelectric electromagnetic composite energy harvester is proposed. Through the experimental study of its output performance, it has been proved that the dynamic magnetic coupling between the PEH and the EEH can reduce the starting wind speed of the energy harvester and increase the total output power of the energy harvester. It can provide a continuous energy supply for more complex and energy-demanding microelectronic components.

(2) In order to further analyze the influence of bistable nonlinear magnetic coupling on the energy capture effect of the MPEGEH, this paper studies the key parameters d_0_ and s_0_ of the MPEGEH through experiments. Experimental research on the effects of key parameters d_0_ and s_0_ on the energy capture efficiency and output characteristics of the MPEGEH found that the initial distance d_0_ between magnet A and B is 37 mm, and the initial distance s_0_ between magnet B and C is 23 mm. The starting wind speed is 28% lower than the CGPEH. The output power of the MPEGEH is much higher than that of the CGPEH after reaching the starting wind speeds. It proves the superiority of the MPEGEH.

(3) Experiments show that when d_0_ and s_0_ are small, the PEH and the EEH both have lower starting wind speeds and higher output characteristics. When the d_0_ and s_0_ are small, the EEH will show strong nonlinearity characteristics, and at a certain wind speed, the phenomenon of a jump will change. Before and after the jump point, the output power of the EEH reaches a peak. When d_0_ is 37 mm, s_0_ is 23 mm, load resistance R_1_ is 7.5 × 10^5^ Ω, R_2_ is 50 Ω, the starting wind speed will be 6.1 m/s, and the PEH, the EEH, and total output power will reach 0.79 mW, 3.92 mW, and 4.71 mW, respectively, under the wind speed of 7.15 m/s. The output power of CGPEH at 11 m/s wind speed is 1.43 mW. At this wind speed, the output power of the MPEGEH is increased by 229% (3.28 mW).

This paper provides a new structural design for the relaxation energy harvester and provides an important experimental basis and reference value in energy collection and nonlinear magnetic coupling.

## Figures and Tables

**Table 1:** Structural and material parameters of the MPEGEH.

Parameter	Value
PZT-5H length (mm), width (mm), and thickness (mm)	32, 12, 0.2
PZT-5H modulus and substrate Yang’s modulus (GPa)	60.6, 106
Dimensions of substrate length (mm), width (mm), and thickness (mm)	120, 30, 0.8
Dimensions of bluff body length (mm), width (mm), and thickness (mm)	80, 60, 60
Dark body quality (g)	80
The quality of magnet A and magnet B (g)	25.5, 25.5
The quality of magnet C and magnet D (g)	8.5, 8.5
Coil internal resistance (Ω)	40.3

## Data Availability

Not applicable.
